# PCI proteins eIF3e and eIF3m define distinct translation initiation factor 3 complexes

**DOI:** 10.1186/1741-7007-3-14

**Published:** 2005-05-17

**Authors:** Chunshui Zhou, Fatih Arslan, Susan Wee, Srinivasan Krishnan, Alexander R Ivanov, Anna Oliva, Janet Leatherwood, Dieter A Wolf

**Affiliations:** 1 Department of Genetics and Complex Diseases, Harvard School of Public Health, 665 Huntington Avenue, Boston, Massachusetts, 02115, USA; 2 Applied Biosystems Inc., Framingham, Massachusetts, USA; 3 Harvard NIEHS Center Proteomics Facility, Harvard School of Public Health, Boston, Massachusetts, USA; 4 Department of Molecular Genetics and Microbiology, State University of New York, Stony Brook, New York, USA; 5 Department of Medicine, Brigham and Women's Hospital, Harvard Medical School, Boston, Massachusetts, USA

## Abstract

**Background:**

PCI/MPN domain protein complexes comprise the 19S proteasome lid, the COP9 signalosome (CSN), and eukaryotic translation initiation factor 3 (eIF3). The eIF3 complex is thought to be composed of essential core subunits required for global protein synthesis and non-essential subunits that may modulate mRNA specificity. Interactions of unclear significance were reported between eIF3 subunits and PCI proteins contained in the CSN.

**Results:**

Here, we report the unexpected finding that fission yeast has two distinct eIF3 complexes sharing common core subunits, but distinguished by the PCI proteins eIF3e and the novel eIF3m, which was previously annotated as a putative CSN subunit. Whereas neither eIF3e nor eIF3m contribute to the non-essential activities of CSN in cullin-RING ubiquitin ligase control, *eif3m*, unlike *eif3e*, is an essential gene required for global cellular protein synthesis and polysome formation. Using a ribonomic approach, this phenotypic distinction was correlated with a different set of mRNAs associated with the eIF3e and eIF3m complexes. Whereas the eIF3m complex appears to associate with the bulk of cellular mRNAs, the eIF3e complex associates with a far more restricted set. The microarray findings were independently corroborated for a random set of 14 mRNAs by RT-PCR analysis.

**Conclusion:**

We propose that the PCI proteins eIF3e and eIF3m define distinct eIF3 complexes that may assist in the translation of different sets of mRNAs.

## Background

Three protein complexes that are conserved from yeast to humans, the 19S proteasome lid, the CSN, and eIF3, contain subunits characterized by two protein motifs: the MPN (Mpr1/ Pad1 N-terminal) and the PCI (proteasome/CSN/eIF3) domains [1]. The proteasome 20S catalytic particle and the 19S regulatory subunit cooperate in degrading polyubiquitylated proteins (reviewed in [2]). The 19S proteasome can be separated into the base complex, which binds and unfolds substrates [3], and the eight subunit lid complex, which cleaves ubiquitin from substrates, thus apparently facilitating the entry of substrates into the catalytic proteasome barrel [4,5].

In higher eukaryotes, the subunits of the 19S lid show pair-wise similarity to the eight subunits of the CSN [6-9]. In vivo, CSN promotes the activity of cullin-RING ubiquitin ligases [10-16], multiprotein complexes containing cullins, the RING protein RBX1, and one of several hundred substrate-specific adaptors [17-22]. The MPN domain containing CSN subunit 5 harbors a protease motif [23] that cleaves the ubiquitin-related peptide NEDD8 from cullins [24,25]. This activity, acting in concert with the CSN-associated deubiquitylation enzyme Ubp12, was proposed to promote cullin function by facilitating the recruitment of labile substrate adaptors [11,16,26,27].

The third PCI/MPN complex, eIF3, is more distantly related to CSN and the 19S lid (reviewed in [28]). Whereas human eIF3 consists of up to 13 subunits, consecutively named eIF3a – l and GA17 [29,30], budding yeast contains only six to eight subunits (depending on purification conditions). Five of these subunits are orthologs of human eIF3a, b, c, g, and eIF3i [31,32] and appear to constitute a conserved core complex [31,33]. Fission yeast contains the same five core subunits, in addition to the non-core subunits eIF3d/Moe1p, eIF3e/Int6p, and eIF3h [34-36]. A putative eIF3f ortholog was also identified, but biochemical evidence confirming it as an authentic eIF3 subunit functioning in protein synthesis is still outstanding [34,35,37,38].

eIF3 is the most complex translation initiation factor and plays at least two important roles in protein synthesis. First, eIF3 binds to the 40S ribosome and facilitates loading of the Met-tRNA/eIF2· GTP ternary complex to form the 43S preinitiation complex. Subsequently, eIF3 apparently assists eIF4 in recruiting mRNAs to the 43S complex. A critical in vivo function of eIF3 core subunits in these processes was indicated by the lethality of the respective budding yeast deletion strains [31]. In contrast, in fission yeast, the non-core subunits eIF3d and eIF3e are not essential for viability or global protein synthesis [34,36,38,39]. It was therefore proposed that distinct subclasses of eIF3 complexes, containing different combinations of core and non-core subunits, may regulate specific subsets of mRNAs in fission yeast [34,36,38,39]. Our study provides the first experimental evidence substantiating this hypothesis by demonstrating that biochemically distinct eIF3 complexes defined by the PCI domain proteins eIF3e and eIF3m (a novel eIF3 protein) associate with different sets of mRNAs.

## Results

### Both *csn6 *and *csn7b *are essential genes

**Figure 1:**
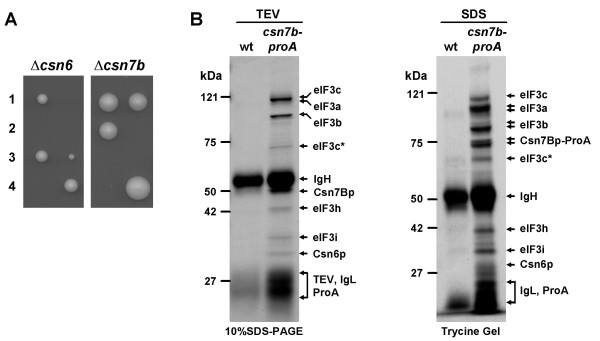
**Analysis of *csn6 *and *csn7b *genes and proteins**. (**A**) The *csn6 *and *csn7b *genes were disrupted in diploid *S. pombe *cells by inserting the *ura4 *marker. Diploids were sporulated and spore viability was examined by tetrad analysis. Only two spores were viable, indicating that *csn6 *and *csn7b *are essential. (**B**) Cell lysate from a strain carrying *csn7b *modified with five C-terminal pro-A tags and a TEV cleavage site at the endogenous genomic locus was absorbed to IgG resin, followed by elution of bound proteins with TEV protease (left panel) or SDS (right panel). Gels were stained with Coomassie Brilliant Blue, and proteins were identified by MALDI-TOF mass spectrometry. The asterisks denote degradation products of elF3c.

CSN complexes of higher eukaryotes typically contain eight distinct subunits (two MPN and six PCI proteins). However, in fission yeast only six subunits are known (Csn1p, 2p, 3p, 4p, 5p, 7Ap; Ref. [13]), none of which are essential for viability [40-42]. We noticed two genes in the *Schizosaccharomyces pombe *genome database, originally annotated as *csn6 *(SPBC4C3.07) and *csn7b *(SPAC1751.03), which encode MPN and PCI domain containing proteins with considerable similarity to metazoan CSN6 and CSN7B, respectively (data not shown). In order to determine whether these genes might function in the known biochemical pathways regulated by CSN, cullin deneddylation and Ubp12p-mediated deubiquitylation [24,26], we deleted *csn6 *and *csn7b *in wild-type diploid cells. Upon sporulation and tetrad analysis, only two viable spores could be recovered in each case, both of which were uracil auxotroph (Fig. 1A, and data not shown). Thus, unlike the genes encoding the six known subunits of the fission yeast CSN [41,42], *csn6 *and *csn7b *are essential.

### Csn6p and Csn7Bp associate with eIF3 components

To elucidate the essential functions of Csn6p and Csn7Bp, we sought to identify their interacting proteins. To this end, we attempted to modify *csn6 *and *csn7b *at their endogenous genomic loci with five consecutive protein A epitope tags (proA). The C-terminal tag also contained a cleavage site for Tobacco Etch Virus (TEV) protease upstream of the protein A moieties. Diploid analysis indicated that C-terminal tagging destroyed the essential function of *csn6 *(data not shown). In contrast, a *csn7b-proA *haploid strain was viable and did not display any obvious phenotypes (data not shown). This indicated that the tagged allele was functional. Total protein lysate from the *csn7b-proA *haploid strain was absorbed to Immunoglobulin G (IgG) resin and specifically retained proteins were eluted with sodium dodecyl sulfate (SDS) or by cleavage with TEV protease. The two eluates were resolved by gel electrophoresis and Csn7Bp-proA interacting proteins were identified by peptide mass fingerprinting using matrix-assisted laser desorption/ionization-time-of-flight (MALDI-TOF) mass spectrometry.

Both eluates contained an identical set of proteins. In addition to Csn6p, the Csn7Bp complex contained the eIF3 core subunits eIF3a, b, c, and i, as well as the non-core subunit eIF3h (Fig. 1B, Table 1). The eIF3g core subunit was not detected using MALDI-TOF in this purification, but was detected in an independent purification of the Csn7Bp complex using the more sensitive method of nano-liquid chromatography and tandem mass spectrometry (LC-MS/MS, see Fig. 5A). However, eIF3e and eIF3d, two previously described components of *S. pombe *eIF3 [34,36,38,39] were consistently absent from the Csn7Bp complex when analyzed either by MALDI-TOF or by LC-MS/MS (Fig. 1B and 5A).

**Figure 2:**
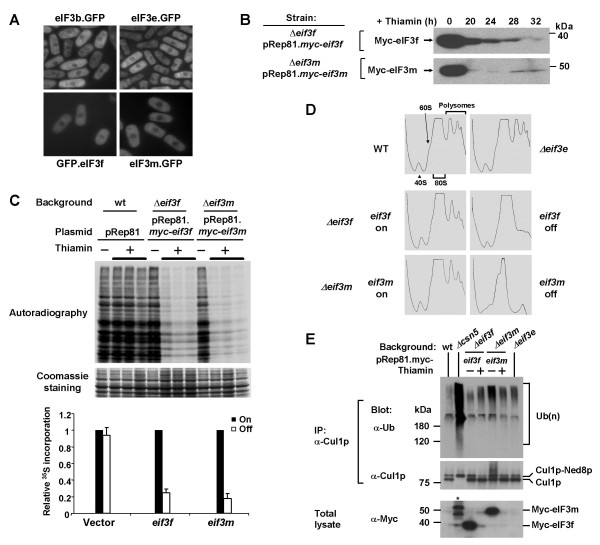
**Roles of *eif3e *and *eif3m*in protein synthesis**. (**A**) Subcellular localization. Live cells expressing GFP-tagged alleles of *eif3b*, *eif3e*, and *eif3m *at the endogenous genomic loci were examined by fluorescence microscopy. N-terminally GFP-tagged *eif3f *was expressed at low levels from the pREP81 plasmid. (**B**) Shut-off strains. Diploid heterozygous *eif3f *and *eif3m *deletion strains were transformed with pREP81 plasmids driving the thiamin-repressible expression of Myc-tagged eIF3f and eIF3m, respectively. Diploids were sporulated and homozygous disruptants carrying the *eif3f *and *eif3m *plasmids were selected. Cells were grown in liquid medium to an OD_595 _of 0.3 in the absence of thiamin, followed by promoter shut-off by the addition of thiamin. Samples were taken at the indicated time points after promoter shut-off and analyzed for the expression of plasmid borne eIF3f and eIF3m by immunoblotting with anti-Myc antibodies. (**C**) Effect on total protein synthesis. The *eif3f *and *eif3m *shut-off strains were maintained in the absence or presence of thiamin as indicated. Strains transformed with empty pREP81 plasmid were included as control. Cells were metabolically labeled with ^35^S-methionine, and aliquots of total cellular proteins were separated by SDS-PAGE and analyzed by autoradiography. The Coomassie Blue stained gel is shown to document equal protein loading. Data were quantified by PhosphorImager analysis and results are displayed in a bar graph. (**D**) Effect on polysomes. Polysome profiles were determined for the indicated strains as described in the Methods section. An *eif3e *deletion strain is shown for reference. (**E**) Effect on CSN-mediated regulation of cullin-RING ubiquitin ligases. Cul1p complexes were immunopurified from the indicated strains and employed in substrate-independent in vitro ubiquitylation reactions with purified E1, the E2 Cdc34p, ubiquitin, and ATP [26]. Polyubiquitin chains formed in the reaction are indicated (top panel). In contrast to *csn5 *mutants, derepression of Cul1p activity is not observed in cells lacking *eif3f*, *eif3m*, or *eif3e*. The neddylation state of Cul1p was determined by immunoblotting (middle panel). Hyperneddylated Cul1p only accumulates in *csn5 *mutants. Shut-off of *eif3f *and *eif3m *expression was verified by immunoblotting with anti-Myc antibodies (lower panel).

Notably, no authentic CSN subunits were found in the Csn7Bp complex, consistent with the previous demonstration that *S. pombe *CSN contains only six non-essential subunits [13]. Structural analysis indicated that Csn6p is not only related to human CSN6, but shows a slightly greater overall similarity to human eIF3f (data not shown). Based on the functional characterization described below (Fig. 2), fission yeast Csn6p therefore appears to be the ortholog of human eIF3f.

A Csn7Bp ortholog is not known in the human eIF3, although the recently identified subunit GA17 [30] shows considerable similarity to Csn7Bp (K. Hofmann, personal communication). In addition, interactions of metazoan CSN7 with eIF3 subunits have previously been observed [43,44]. With regards to the unified nomenclature of eIF3 subunits [29], we propose eIF3m as a more appropriate name for the novel fission yeast eIF3 subunit encoded by *csn7b *(see Table 1). Throughout this manuscript, we will use the proposed new names for the *eif3f *and *eif3m *genes and their products. The original annotations have recently been revised by the curator of the *S. pombe *genome database, in order to reflect the findings presented here.

### The eIF3f and eIF3m proteins are primarily cytoplasmic

The evidence presented above suggested that eIF3f and eIF3m are essential subunits of *S. pombe *eIF3. To substantiate this possibility, we determined the subcellular localization of eIF3f and eIF3m. If these proteins were authentic eIF3 subunits involved in protein synthesis, they should assume a subcellular localization similar to known eIF3 subunits. We therefore prepared three strains with green fluorescent protein (GFP) tagged alleles of eIF3b, eIF3e, and eIF3m integrated into their respective genomic loci. In addition, since C-terminal GFP-tagging destroyed the essential function of the small eIF3f protein, we prepared a strain mildly overexpressing N-terminally GFP-tagged eIF3f from a pREP81 plasmid. Fluorescence microscopy of live cells revealed that all four proteins were primarily localized in the cytoplasm (Fig. 2A), which would be consistent with a function in protein translation. In contrast, CSN subunits are largely nuclear in fission yeast ([41], and our own unpublished observation).

### eIF3f and eIF3m are required for global protein synthesis

To directly assess the potential role of eIF3f and eIF3m in protein synthesis, we prepared haploid *eif3f *and *eif3m *deletion strains maintained viable by plasmids driving the regulated expression of *eif3f *and *eif3m*, respectively. Expression from these plasmids could be turned off by the addition of thiamin to the growth media, resulting in a strong reduction in protein levels within 20 to 32 hours (Fig. 2B). Coincident with shut-off of eIF3f and eIF3m expression, global cellular protein synthesis was diminished by ~80%, as determined by metabolic labeling with ^35^S-methionine (Fig. 2C).

Further biochemical analysis by sucrose gradient velocity centrifugation revealed a strong reduction in the formation of polysomes in *eif3f *and *eif3m *mutants following promoter shut-off (Fig. 2D). In contrast, as described previously [34,36,38,39], *eif3e *mutants showed only a minor reduction in total protein synthesis (data not shown) and essentially normal polysomes (Fig. 2D).

The conserved cullin deneddylation function of CSN was not impaired either in *eif3f *and *eif3m *mutants or in *eif3e *mutants, since cullin 1 (Cul1p) did not accumulate exclusively in the neddylated form as it does in *csn5 *deletion strains (Fig. 2E). Similarly, unlike in *csn5 *mutants (Fig. 2E; [26]), CSN/Ubp12p-mediated inhibition of Cul1p in vitro ubiquitin ligase activity was not impaired upon shut-off of either *eif3f *or *eif3m *expression. Cul1p neddylation and activity were also unaffected in *eif3e *mutants (Fig. 2E). These data suggested that eIF3f and eIF3m are bona fide subunits of fission yeast eIF3 but not CSN, performing essential functions in protein synthesis similar to eIF3 core subunits.

### The eIF3m and eIF3e proteins define distinct eIF3 complexes

Although the purification of the eIF3m complex robustly retrieved roughly stoichiometric amounts of known eIF3 subunits, two subunits, eIF3d and eIF3e, were conspicuously absent (Fig. 1B, Table 1). To exclude the possibility that we had overlooked these subunits because they were obscured by the IgG bands, we affinity-purified eIF3e-associated proteins from an *eif3e-proA *strain. The retrieved proteins were identified using MALDI-TOF mass spectrometry as before.

**Figure 3:**
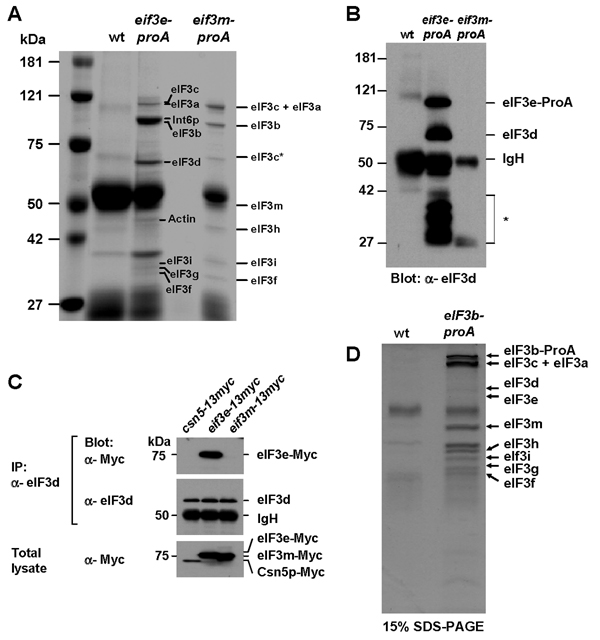
**Distinct eIF3 complexes**. (**A**) eIF3 complexes associated with eIF3e and eIF3m were affinity-purified by absorption of the respective pro-A-tagged proteins to IgG beads. Bound proteins were eluted by cleavage with SDS (eIF3e) or TEV protease (eIF3m). (**B**) The eluates described in (A) were analyzed by immunoblotting with eIF3d antibodies. The asterisk refers to cross-reactivity of the secondary antibody with Ig light chains. (**C**) Lysates from cells expressing Myc-tagged Csn5p, eIF3e, and eIF3m from their endogenous genomic loci were immunoprecipitated with anti-eIF3d antisera. Coprecipitated proteins were identified by immunoblotting as indicated. Total cell lysates are shown to document expression levels of the endogenously tagged proteins. (**D**) Protein lysate from cells expressing protein A-tagged eIF3b from the endogenous promoter was absorbed to IgG beads, and specifically retained proteins were identified by LC-MS/MS.

Like the eIF3m complex, the eIF3e complex contained roughly stoichiometric amounts of the eIF3 core subunits a, b, c, g, and i (Fig. 3A, Table 1). In addition, eIF3f was present while, unexpectedly, eIF3m and eIF3h were missing. We did not detect the proteasome lid subunit Rpn5p, which previously had been shown to associate with eIF3e and eIF3d [45]. This observation indicated that the described eIF3d/eIF3e/Rpn5p interaction is either unstable under our purification conditions or contains only a minor fraction of the total eIF3e engaged in protein interactions.

These results raised the intriguing possibility that fission yeast contains two distinct eIF3 complexes that comprise an overlapping set of core subunits, but are distinguished by the presence of either the essential PCI protein eIF3m and the MPN domain protein eIF3h or the non-essential PCI protein eIF3e and its binding partner eIF3d. To exclude the possibility that our mass spectrometry analysis missed substoichiometric amounts of eIF3d in the eIF3m complex, we analyzed eIF3m complexes by immunoblotting with eIF3d antisera. Whereas eIF3d was readily detectable in the eIF3e complex, it was undetectable in the eIF3m complex (Fig. 3B). In the reciprocal experiment, eIF3d antibodies co-immunoprecipitated eIF3e-13Myc, but not eIF3m-13Myc or Csn5p-13Myc (Fig. 3C).

To exclude the possibility that our cell lysis or affinity purification conditions led to uncontrolled release of eIF3 subunits, thus mimicking the existence of distinct subcomplexes, we performed a purification using the protein A-tagged eIF3b core subunit as bait. As determined by LC-MS/MS performed on excised bands detected in the complex upon PAGE separation, the eIF3b complex contained all known eIF3 core components as well as eIF3f and eIF3m (Fig. 3D). The eIF3e and eIF3d proteins were also present, albeit in clearly substoichiometric amounts. These findings indicate that purification of eIF3b resulted in the copurification of the distinct eIF3m- and eIF3e-containing complexes. Taken together, these data strongly suggested the existence of two distinct eIF3 complexes defined by the PCI proteins eIF3m and eIF3e.

### The eIF3m and eIF3e proteins associate with distinct sets of mRNAs

The finding that *eif3m *is essential, whereas *eif3e *is dispensable, suggested the possibility that the different eIF3 complexes they define regulate different subsets of mRNAs. To approach this, we sought to identify mRNAs specifically associated with the eIF3m and eIF3e complexes. The complexes were affinity-purified as described above in the presence of RNAse inhibitors, and the associated RNA was extracted, amplified, and converted to Cy3-labeled cDNA as described in the Methods section. The Cy3-labeled cDNA was hybridized competitively with Cy5-labeled cDNA prepared from total RNA onto microarrays representing all 4988 predicted *S. pombe *open reading frames (ORFs) and RNAs. Based on the background subtracted hybridization signals, eIF3-associated mRNAs were ranked according to their factor of enrichment in the eIF3m and eIF3e complexes over a mock purified sample ([see additional file 1]).

**Figure 4:**
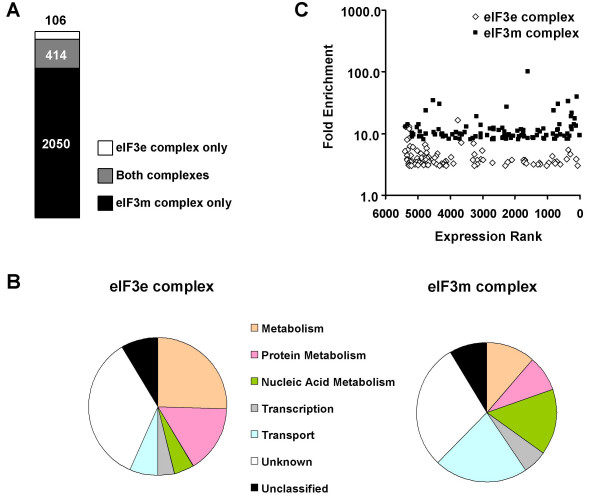
**Association of mRNAs with eIF3 complexes**. (**A**) Messenger RNAs associated with eIF3e and eIF3m complexes were identified by microarray hybridization as described in the Methods section. The graph indicates the numbers of mRNAs enriched more than three-fold over mock in both complexes. (**B**) Classification into functional groups of the 106 mRNAs most highly and uniquely enriched in complexes with eIF3e or eIF3m. (**C**) The relative expression ranks of the 106 most highly and uniquely enriched mRNAs in the eIF3e and eIF3m complexeswere determined using the relative hybridization signals obtained with total *S. pombe *cDNA ([see additional file 1]). The expression ranks were blotted against the factor of enrichment of each mRNA in the eIF3 complexes. No correlation between expression rank and enrichment is apparent, indicating that highly expressed mRNAs were not unspecifically enriched in eIF3 complexes. The 5407 individual features on the microarray slides represent the 4988 ORFs and transcripts predicted in the Sanger Centre *S. pombe *genome database and various controls. To simplify the analysis, expression ranks were also assigned to these control spots, thus resulting in expression ranks for some ORFs higher than the theoretically possible number of 4988.

The microarray analysis suggested a global role for eIF3m and a more restricted function of eIF3e in translation. Using an arbitrary cut-off value of three-fold enrichment over mock, eIF3m associated with 2464 different mRNAs, whereas eIF3e associated with only 520 distinct species (Fig. 4A). We observed 414 mRNAs enriched more than three-fold in both samples. (Fig. 4A). In addition, our analysis revealed 106 mRNAs uniquely enriched in the eIF3e complex, whereas 2050 transcripts were uniquely enriched in the eIF3m complex (Fig. 4A,C).

A ranked list of all 106 mRNAs that were uniquely enriched more than three-fold in the eIF3e complex was assembled ([see additional file 2]). Approximately 75% of these mRNAs fell into one of three categories: mRNAs encoding proteins involved in intermediary metabolism (25.5%), those encoding proteins involved in protein metabolism (14.2%), and those encoding proteins with unknown functions (35.8%; Fig. 4B). The remainder was distributed roughly equally among four categories: mRNAs encoding proteins involved in nucleic acid metabolism, those encoding proteins involved in transcription, those encoding transporter proteins, and those encoding unclassified proteins.

A corresponding list of the 106 most highly enriched mRNAs exclusively present in the eIF3m complex (out of a total of 2050 enriched by a factor greater than three) revealed that ~58% also fell into three categories: mRNAs encoding proteins involved in nucleic acid metabolism (9.4%), those encoding transporter proteins (18.9%), and those encoding proteins of unknown function (20.8%). The remainder was roughly equally distributed among four categories: mRNAs encoding proteins involved in intermediary metabolism, those mRNAs encoding proteins involved in protein metabolism, those encoding proteins involved in transcription, and those encoding unclassified proteins (Fig. 4B, [see additional file 2]).

To exclude the possibility that our affinity purification protocol unspecifically enriched highly expressed mRNAs, we averaged the data from the three competively hybridized samples of total *S. pombe *cDNA to determine the relative expression rank of each mRNA (rank 1 = most abundant mRNA, rank 5407 = least abundant mRNA; see legend to Fig. 4C for detail). A plot of expression rank vs. factor of enrichment of the 106 mRNAs most enriched in the eIF3e and eIF3m complexes revealed that the eIF3m complex contained mRNAs that were distributed equally over the entire range of expression levels. In contrast, the eIF3e complex was enriched for rare mRNAs (~65 % of eIF3e-associated mRNAs had an expression rank > 4000; see Fig. 4C). The lack of a linear correlation between mRNA expression level and enrichment in the eIF3e and eIF3m complexes argues against unspecific copurification of abundant mRNAs.

### Specific association of mRNAs with eIF3m and eIF3e complexes

**Figure 5:**
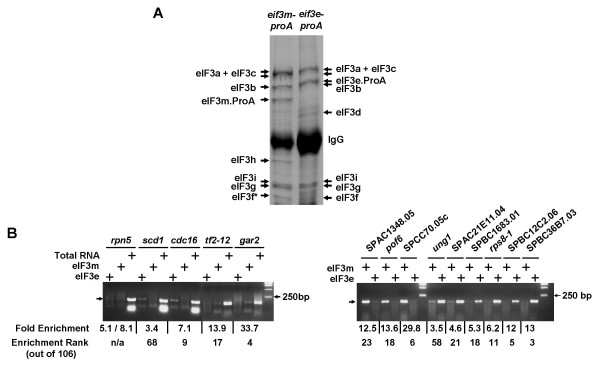
**Specific association of mRNAs with the eIF3e and eIF3m complexes**. (**A**) The eIF3 complexes shown were affinity-purified as described in the Methods section, and separated by SDS-PAGE followed by staining with Coomassie Brilliant Blue. The labeled bands were identified by LC-MS/MS. eIF3f* refers to a band in the eIF3m complex that was not identified by mass spectrometry in this experiment, and is therefore assigned based on its comigration with eIF3f identified in the eIF3e complex (right lane). eIF3f was positively identified by MALDI-TOF mass spectrometry as a subunit of the eIF3m complex in Fig. 1B. (**B**) The indicated eIF3e- and eIF3m-associated mRNAs were extracted from the purified complexes and employed in RT-PCR reactions using primers specific for each mRNA (see Table 3). PCR products obtained with total *S. pombe *RNA are shown for reference in the left panel. The factor of enrichment of each mRNA in the respective eIF3 complex and the enrichment rank (out of all 106 mRNAs enriched in either complex) are indicated below the gels.

To validate the results from the microarray hybridizations, we chose several mRNAs at random from each list of 106 and used RT-PCR to confirm their interactions with the respective eIF3 complexes. eIF3e-proA- and eIF3m-proA-associated complexes were affinity-purified as before and separated by PAGE. Both complexes showed the same differential subunit composition as described above (Fig. 5A). The associated RNA was purified, split into equal portions, and employed in RT-PCR reactions with gene-specific primer pairs.

The RT-PCR analysis of a total of fourteen mRNAs included eight mRNAs identified in the eIF3e complex, five mRNAs identified in the eIF3m complex, and one mRNA found to be associated with both complexes with similar enrichment factors. These mRNAs varied widely in their factors of enrichment and their enrichment ranks as indicated in Fig. 5B. Without exception, all eight mRNAs found to be enriched in the eIF3e complex by microarray analysis were also found preferentially associated with this complex by RT-PCR (Fig. 5B). Based on this result, we calculate a false positive rate of less than 0.11 with a 95% confidence interval of 0.00 – 0.31. Whereas mRNAs with a high enrichment factor (>10, [see additional file 2]) appeared to be exclusively associated with eIF3e-proA (within the limit of detection), those with a lower enrichment factor (<10) also displayed a low amount of association with the eIF3m complex (Fig. 5B). Conversely, all five mRNAs highly enriched in the eIF3m complex as determined by microarray analysis ([see additional file 2]) were almost exclusively bound to this complex when analyzed by RT-PCR (Fig. 5B).

Notably, the mRNA encoding the proteasome subunit Rpn5p, which was found in both complexes by microarray analysis with similar enrichment factors (eIF3e = 5.1; eIF3m = 8.1) was also confirmed as being associated with both eIF3 complexes by RT-PCR (Fig. 5B). These results strongly suggest that the eIF3m and eIF3e complexes specifically associate with distinct sets of mRNAs.

## Discussion

### PCI proteins define distinct eIF3 complexes

Our results show that Csn6p and Csn7Bp are subunits of fission yeast eIF3. Csn6p was shown to be the eIF3f subunit conserved in all eukaryotes except budding yeast. Consistent with this finding, eIF3f has previously been found to be associated with eIF3g, although the role of eIF3f in protein synthesis had not been examined [34]. Our study further identified two distinct eIF3 complexes defined by the PCI proteins eIF3e and eIF3m (Fig. 3A). No direct equivalent of eIF3m is apparent in eIF3 preparations from other eukaryotes. Considering the essential function of eIF3m in protein synthesis in fission yeast, it is possible that other PCI proteins substitute for eIF3m in other organisms.

Consistent with this idea is the finding that the PCI domain containing GA17, a recently identified protein copurifying with human eIF3 [30], shows more similarity to eIF3m than to any other PCI protein (K. Hofmann, personal communication). In addition, the PCI domain containing CSN subunits 1, 3, 7, and 8 were found to interact with eIF3 subunits in *Arabidopsis thaliana *and in human cells [43,44,46]. In addition, Pci8p/Csn11p, a non-essential PCI subunit of the budding yeast CSN [47,48], copurifies with essential eIF3 core subunits [49]. Although the functional significance of these interactions is still unclear, our findings raise the intriguing possibility that they define multiple subclasses of distinct eIF3 complexes. Whereas fission yeast appears to rely on an essential eIF3 complex classified by eIF3m and a non-essential complex specified by eIF3e, higher eukaryotes may divide the task of mRNA translation among a multitude of eIF3 complexes. Since the individual PCI proteins defining these subcomplexes are expected to be present in substoichiometric amounts relative to core components, as is eIF3e in the *S. pombe *core complex, they may have escaped detection in bulk eIF3 preparations from higher eukaryotes.

### Distinct eIF3 complexes and translational specificity

Although our finding of distinct eIF3 complexes that associate with different sets of mRNAs was surprising, circumstantial evidence pointing to their existence was provided in previous studies. For example, fission yeast eIF3e copurifies with eIF3 core subunits, and yet, unlike core subunits, eIF3e is not required for global protein synthesis or viability. Two competing models were proposed to explain the pleiotropic phenotype of *eif3e *deletion mutants, which includes slow growth in minimal media and meiotic defects [34,36,38,39,50,51]. In the first model, eIF3e regulates the translation of all transcripts, but rare mRNAs may be more affected by a fractional reduction in translation efficiency and thus give rise to distinct phenotypes. In the second model, eIF3e regulates the translation of a specific subset of mRNAs encoding proteins whose depletion leads to distinct phenotypes but not lethality. The findings presented here strongly favor the second model.

Our microarray analysis revealed 106 mRNAs uniquely enriched in the eIF3e complex, many being rare, whereas 2050 transcripts were uniquely enriched in the eIF3m complex. The latter finding is consistent with the severe protein synthesis defect and lethality of *eif3m *deletion mutants. Whereas these mRNA lists were based on an arbitrary cut-off value of three-fold enrichment over mock, this criterion proved stringent enough to confirm specific association with their respective eIF3 complexes of all 14 mRNAs retested by RT-PCR. Thus, since we calculate the false positive rate to be less than 0.11 with 95% confidence interval 0.00 – 0.31, the maximum number of mRNAs listed potentially false positively as enriched in the eIF3e complex would be 33 out of 106 (with 95% confidence).

However, we consider it unlikely that all mRNAs found enriched in the eIF3e complex are exclusive translational targets of this complex, because the cellular phenotypes of *eif3e *mutants are rather discrete. Although many of the eIF3e-associated mRNAs encode essential proteins, viability, global protein synthesis, and polysome formation are not affected in these mutants when they are grown in rich media (Fig. 2D, and data not shown). It is therefore likely that eIF3m or other PCI domain proteins can deputize for eIF3e in many cases. In fact, our microarray study revealed that a substantial portion of eIF3e-associated mRNAs was also recovered in the eIF3m complex (Fig. 4A). Nonetheless, we strongly suspect that inefficient translation of a limited set of eIF3e-associated mRNAs contributes to distinct cellular phenotypes found in *eif3e *mutants (see below).

### The eIF3e protein and translational control during stress response

The *eif3e *mutants display marked sensitivity to a wide variety of cellular stresses, such as osmotic stress, nutrient starvation, and low temperature [34,38,39,50,52] In addition, *eif3e *mutants show synthetic lethality with proteasome mutants, which are presumed to accumulate a large load of misfolded proteins [45]. Conversely, overexpression of *eif3e *confers resistance to a broad spectrum of unrelated drugs, whose only commonality appears to be that they induce cellular stress [38]. Overexpression of eIF3e also activates genes involved in stress defense, such as thioredoxin [38]. Interestingly, the mRNA encoding thioredoxin was also identified as a putative translational target of eIF3e ([see additional file 2]). Finally, eIF3e undergoes relocalization into cytoplasmic foci in response to heat and osmotic stress, thus implicating it in stress regulation [35].

Global protein synthesis is actively switched off in response to cellular stress and nutrient deprivation (reviewed in Refs. [53,54]). This shut-off protects cells from proteotoxicity by relieving chaperones of their load of unfolded client proteins, conserving amino acids for other essential functions, and attenuating the metabolic consequences of protein synthesis. However, long-term adaptation to stress conditions, repair and recovery require synthesis of new stress-induced proteins. During the stress response, some mRNAs must be translated in the presence of a repressed general translation machinery, suggesting that select eIFs can escape the global repression. Our studies raise the intriguing possibility that the eIF3e complex has such a specialized function during stress response. Lack of *eif3e *may prevent stress-induced synthesis of its critical translational targets, thus leading to the known stress sensitivity of the mutant. Substantial additional work will be required to address this possibility.

### Potential mechanisms underlying mRNA discrimination by eIF3 complexes

Several lines of evidence have begun to implicate other general translation factors, including eIF2 and eIF4, in translational specificity [55]. Importantly, these factors are downstream of important signaling cascades, including the nutrient-sensing TOR pathway and a variety of stress-induced kinases (reviewed in Refs. [55,56]). Although the details of these regulatory mechanisms are beginning to emerge, it is still unclear exactly how eIF2 and eIF4 contribute to mRNA specificity.

Since eIF3 cooperates with the cap-binding eIF4 complex in directing the 5' end of mRNAs to the 40S ribosome, and several eIF3 subunits contain RNA binding motifs, our data is consistent with a model in which eIF3 complexes defined by distinct PCI proteins recognize determining features of mRNAs presented to the 40S subunit in concert with eIF4. In agreement with this idea is the recent finding that the human PCI protein eIF3a specifically facilitates the translation of the mRNA encoding ribonucleotide reductase M2 [57]. In addition, *A. thaliana *eIF3h, a non-core subunit, was recently shown to be specifically required for efficient translation of the transcription factor ATB2 [46].

While we have so far been unsuccessful in identifying common sequence elements within the upstream regions of mRNAs enriched in the eIF3e complex (data not shown), this analysis was severely hampered by the fact that 5'UTRs are poorly defined in *S. pombe*. It is therefore still possible that critical features, including secondary structure determinants, lie within the 5'UTR or other regions of eIF3e-associated mRNAs. Substantial additional work will be required to delineate the exact mechanism of how eIF3e may contribute to the translation of specific mRNAs and the potential impact of this mechanism on human cancers defective in INT6/eIF3e [58,59].

## Conclusion

We provide biochemical evidence for the existence of two distinct eIF3 complexes in fission yeast. These complexes contain an overlapping set of subunits, but are distinguished by the PCI proteins eIF3e and eIF3m. Based on the finding that the distinct eIF3 complexes associate with different mRNAs, we propose that they have different translational specificities.

## Methods

### Fission yeast techniques

Preparation of fission yeast cultures, cDNA cloning, yeast transformation, PCR-based genomic epitope tagging and gene deletion, mating, and tetrad analysis were carried out using standard methods as described [26]. The strains used in this study are listed in Table 2.

### Affinity purification and mass spectrometry

Epitope-tagged strains (*eif3m-proA *and *eif3e-proA *strains) were grown in 12 L yeast extract and supplements (YES) medium to 1.5 optical density (OD) at 595nm wavelength and harvested by centrifugation. Cell lysates were prepared by bead lysis in a lysis buffer containing 50 mM Tris-HCl pH 8.0, 150 mM NaCl, 0.5% Triton X-100, 1 mM dithiothreitol, supplemented with protease inhibitors (10 ug/ml leupeptin, 10 ug/ml pepstatin, 5 ug/ml aprotinin and 1mM phenylmethylsulfonylfluoride). Upon centrifugation at 17,000 rpm for 50 min at 4°C, 300 mg total protein in a volume of 50 ml was absorbed to 100 ul Dyna beads (Dynal Biotech, Oslo, Norway) coupled to whole rabbit IgG (Jackson Immunochemicals, West Grove, Pennsylvania, USA). Mock purifications were performed in parallel by absorbing lysate from cells lacking any tagged proteins to IgG beads. Beads were collected by a magnetic device and washed five times in 5 ml lysis buffer. TEV protease (Invitrogen, Carlsbad, California, USA) cleavage or elution in 5% SDS solution were used to collect bound proteins. Eluted proteins were separated on trycine gels or by conventional SDS-polyacrylamide gel electrophoresis (SDS-PAGE), and visualized by Coomassie G250 staining. Protein bands were excised from gels, digested with trypsin (Promega Corporation, Madison, Wisconsin, USA), and subjected to mass spectrometry. The eIF3 complexes were analyzed by MALDI-TOF, and by LC-MS/MS at the Harvard NIEHS Center Proteomics Facility, according to standard procedures.

### Protein synthesis and polysome analysis

The *eif3f *and *eif3m *conditional shut-off strains were inoculated in 10 ml Edinburgh Minimal Media (EMM) with or without thiamin and cultured at 30°C for 30 hrs. 100 uCi ^35^S-methionine were added to the cells, which were labeled for 30 min and then harvested by centrifugation. Cell lysates were prepared by bead disruption and equal amounts of the lysates were analyzed by SDS-PAGE. ^35^S-methionine incorporation was determined by autoradiography and quantified by PhosphorImager analysis (Bio-Rad, Hercules, California, USA).

Polysome analysis was performed exactly as described in Ref. [60]. Briefly, cells were grown in EMM with or without thiamin for 30 hrs. Cycloheximide (100 ug/ml) was added to each culture for 20 min. Cells were harvested and lysed in breaking buffer (20 mM Tris/HCl pH 7.4, 50 mM NaCl, 30 mM MgCl_2_, 1 mM DTT, 50 ug/ml cycloheximide, 0.2 mg/heparin, 50 U/ml SuperRNasein, and protease inhibitors), followed by centrifugation at 13,000 × g for 5 min. 20 A_260 nm _units of each supernatant in a volume of 500 ul were fractionated on 5-45 % sucrose gradients for 3 hrs at 30,000 rpm using a SW40-Ti rotor in a Beckman L70 centrifuge. Polysome profiles were obtained by monitoring the absorbance at 254 nm along the gradient using a Bio-Rad fractionator, and the output was recorded using a Bio-Rad UV detector.

### RT-PCR

RNAs associated with the eIF3e and eIF3m complexes were purified as described above. Primer design and RT-PCR conditions were according to the manual supplied with the Platinum Quantitative RT-PCR Thermoscript kit (Invitrogen). Primers used for RT-PCR are listed in Table 3. The primer concentration for actin amplification was 0.25 uM. Other primers were used at 1 uM. Total RNA was obtained as described [19].

### Immunological techniques

Immunoprecipitation, immunoblotting, and in vitro ubiquitination assays were carried out exactly as described previously [26]. Antibodies against protein A and ubiquitin were purchased from Sigma and Genzyme (Cambridge, Massachusetts, USA), respectively. Anti-eIF3d/Moe1p polyclonal antibody was provided by E. Chang (Baylor College of Medicine, Houston, Texas, USA)

### Microarray analysis

The eIF3 complexes were affinity-purified as described above, except that 50 U/ml SuperRNasein (Ambion, Austin, Texas, USA) and 5 mM ribonucleoside vanadyl complex (New England Biolab, Beverly, Massachusetts, USA) were added to the lysis buffer. A mock purification was performed under identical conditions with lysate from cells lacking any tagged proteins. The methods for extraction of RNA, cDNA synthesis, RNA amplification, and aminoallyl labeling were adopted from previous publications [61,62]. Briefly, after binding and washing, beads were treated with 3 U/ul DNase I, followed by elution with 5 % SDS, extraction with phenol/chloroform and then ethanol precipitation. The associated RNA was reversely transcribed using Superscript II reverse transcriptase (Gibco-BRL Division of Invitrogen) with 1 uM oligo-dT(15)-T7 primer (5'-AAA CGA CGG CCA GTG AAT TGT AAT ACG ACT CAC TAT AGG CGC-3') and 1 uM template switch primer (5'-AAG CAG TGG TAT CAA CGC AGA GTA CGC GGG-3'). Full-length double stranded (ds)-cDNA was synthesized using Advantage Polymerase (Clontech, BD Biosciences, Mountain View, California, USA). Newly synthesized ds-cDNA was passed through Bio-6 columns (Bio-Rad) and dried. RNA amplification was performed with the T7 megascript kit (Ambion). Amplified RNA was purified using the RNeasy kit (Qiagen, Hilden, Germany) and reversely transcribed with Superscript II reverse transcriptase in the presence of aminoallyl-dUTP in reactions primed with oligo-dT (Invitrogen). After RNAse H digestion and purification on QIAquick columns (Qiagen), the aminoallyl-cDNA was labeled with Cy3, and purified on QIAquick columns. Control Cy5-labeled aminoallyl-cDNA was made by oligo dT primed reverse transcription of total *S. pombe *RNA.

Labeled cDNAs were hybridized onto glass slide microarrays. Microarrays were made by spotting purified ds-PCR products 500 to 1200 bp in length onto glass slides coated with aminopropylsilane (Erie Scientific, Portsmouth, New Hampshire, USA). The PCR products used represented 4988 predicted ORFs and transcripts as annotated by the Sanger Centre [63] and are located at the 3' end of predicted genes to maximize sensitivity in detecting oligo-dT primed reverse transcription products. Descriptions of primer pairs and the features on the microarrays are available at the Longhorn Array Database [64].

Median pixel intensities for each spot obtained from hybridizations of eIF3-associated RNA ([see additional file 1, sheet 1]) were corrected by local background subtraction and divided by the corresponding values obtained from a mock purification. This resulted in the factor of enrichment of mRNAs bound to eIF3 complexes over mock. The mRNAs were ranked according to their factor of enrichment ([see additional file 1, sheet 2]). An enrichment factor of three was chosen as an arbitrary threshold. We found 2464 mRNAs were enriched greater than three-fold over mock in the eIF3m complex, while we observed 520 mRNAs enriched in the eIF3e complex ([see additional file 1, sheet 3]). Comparison of the data sets revealed 414 mRNAs that occurred in both complexes, 2050 mRNAs uniquely enriched in the eIF3m complex and 106 mRNAs that were exclusively enriched in the eIF3e complex ([see additional file 1, sheet 4; additional file 2]). A corresponding list of the 106 mRNAs most highly and uniquely enriched in the eIF3m complex was assembled ([see additional file 2]) and used to select specific mRNAs for RT-PCR analysis.

The competitive Cy5-labeled total cDNA provided clear microarray feature identification and a guide to relative sequence abundance in the pre-purification sample from which the relative expression rank of each mRNA was determined ([see additional file 1, sheet 2]). In this study, data were not adjusted by ratio normalization to total RNA signals or local area normalization functions. The microarray data were deposited with the public ArrayExpress (E-MEXP-208) [65] and GEO [66] databases.

## List of abbreviations

CSN COP9 signalosome

Cul Cullin

eIF Eukaryotic initiation factor

EMM Edinburgh Minimal Media

GFP Green fluorescent protein

IgG Immunoglobulin G

LC MS/MS Liquid chromatography tandem mass spectrometry

MALDI-TOF Matrix-assisted laser desorption ionization time-of-flight

MPN Mpr1/Pad1 N-terminal

OD Optical density

PAGE Polyacrylamide gel electrophoresis

PCI Proteasome, COP9 Signalosome, eIF3

ProA Protein A

RT-PCR Reverse transcription-polymerase chain reaction

TEV Tobacco etch virus

YES Yeast extract and supplements (growth media)

## Authors' contributions

CZ performed the bulk of the experiments shown in this study. FA and SW prepared various strains used in this study. SK and ARI performed mass spectrometry. AO performed the microarray hybridizations, and JL participated in the design of the microarray studies and in data analysis. DAW contributed to study design and drafting of the manuscript.

## Supplementary Material

Additional File 1 This article is accompanied by a Microsoft Excel file (.xls) containing microarray data (see Methods for description). Click here for file

Additional File 2 A Microsoft Excel file (.xls) is included containing the lists of the 106 mRNAs most highly and uniquely enriched in the eIF3e and eIF3m complexes. Click here for file

## Figures and Tables

**Table 1:** Summary of eIF3 subunits in various eukaryotes

***Unified Nomenclature***	***Domain***	***Human***	***S. cerevisiae (Core subunits)***	***S. pombe Csn7Bp complex***	***S. pombe Int6p complex***	***S. pombe eIF3b complex***
eIF3a	PCI	p170	Tif32p	p107	p107	p107
eIF3b	RRM	p116	Prt1p	p84	p84	p84
eIF3c	PCI	p110	Nip1p	p104	p104	p104
eIF3d	-	p66	-	-	Moe1p	Moe1p
eIF3e	PCI	p48	-	-	Int6p	Int6p
eIF3f	MPN	p47	-	Csn6p	Csn6p	Csn6p
eIF3g	RRM/Zn finger	p44	Tif35p	Tif35p	Tif35p	Tif35p
eIF3h	MPN	p40	-	p40	-	p40
eIF3i	WD repeat	p36	Tif34p	Sum1p	Sum1p	Sum1p
eIF3j	-	p35	-	-	-	-
eIF3k	PCI	p28	-	-	-	-
eIF3l	PCI	p67	-	-	-	-
eIF3m	PCI	GA17 (?)	-	Csn7Bp	-	Csn7Bp

**Table 2:** Strains used in this study

**Name**	**Genotype**		**Source**
DS448/1	*leu1-32 ura4-d18 ade6-704*	*h+*	Lab stock
DS448/2	*leu1-32 ura4-d18 ade6-704*	*h-*	Lab stock
C399/3	*leu1-32 ura4-d18 ade6-704*	*csn5.13myc kan h-*	Lab stock
C485/3	*leu1-32 ura4-d18 ade6-704*	*eif3m.13myc kan h-*	Lab stock
C617/1	*leu1-32 ura4-d18 ade6-704*	*eif3m.CBP.tev.5proA kan h-*	Lab stock
C642/1	*leu1-32 ura4-d18*	*diploid, eif3f/eif3f::ura4*	This study
C642/2	*leu1-32 ura4-d18*	*diploid, eif3f/eif3f::ura4*	This study
C648	*leu1-32 ura4-d18 ade6-704*	*eif3e.cbp.tev.5proA kan h+*	This study
C650/1	*leu1-32 ura4-d18 ade6-704*	*eif3f::ura4 pRep81.6xhis.myc.eif3f h-*	This study
C650/2	*leu1-32 ura4-d18 ade6-704*	*eif3m::ura4 pRep81.6xhis.myc.eif3m h-*	This study
C652/1	*leu1-32 ura4-d18 ade6-704*	*eif3e::ura4 h+*	This study
C663/1	*leu1-32 ura4-d18 ade6-704*	*eif3m.gfp kan h-*	This study
C663/2	*leu1-32 ura4-d18 ade6-704*	*eif3e.gfp kan h-*	This study
C665	*leu1-32 ura4-d18 ade6-704*	*eif3e.13myc kan h-*	This study
C701	*leu1-32 ura4-d18 ade6-704*	*eif3b.gfp kan, h-*	This study
C702	*leu1-32 ura4-d18 ade6-704*	*eif3b.cbp.tev.5proA kan h-*	This study

**Table 3:** RT-PCR primers

**Gene Name**	**Sequence**	**Product Size (bp)**
*actin*F	GAGCTTCCTGATGGTCAAGT	200
*actin*R	GGATACATAGTGGTACCACC	
*cdc16*F	AAGTTAGCGCCCAAATCACC	150
*cdc16*R	TTTTGAATGCCCCCCCACGG	
*gar2*F	GGTGCCATTGAGAAACCTTC	150
*gar2*R	CCGAACCTTCAAAGAAATCA	
*pof6*F	TCTGATCGGCCTAAGCTGTC	150
*pof6*R	CAATCTTGCAAATTCAACTC	
*rpn5*F	TGAGAAGCAAGTTCGTCAGG	160
*rpn5*R	TGAAACAATCGATGACAAGT	
*rps8*-1F	AAGCGTATTCACGAGGTCCG	150
*rps8*-1R	CAACTCGTTGTTAGAAGGGT	
*scd1*F	TCAGAGTTGGCTGCTTTCTT	150
*scd1*R	ATCCATTGTGTGCCCTGTTC	
SPAC1348.05F	CTTAGTGAACAGTTTGGAAG	150
SPAC1348.05R	TGATAAACCAACGGATCCGA	
SPAC21E11.04F	GAGACATCACCTGCTCCAGA	150
SPAC21E11.04R	TTTGCTCCGGTGACTAGGTG	
SPBC12C2.06F	TCTGTTCCCAAACCTCAAGC	150
SPBC12C2.06R	ATCGATTTTTGCACCTTTAG	
SPBC1683.01F	GGCCGTAAATTTGTCTACGG	150
SPBC1683.01R	ACCACCAATACCAACACCAA	
SPBC36B7.03F	CTCTCAATTAAATTTCACCC	150
SPBC36B7.03R	AGGAGTACCGTATAAAGCAT	
SPCC70.05cF	GTACCTGGAAATAACTCTCC	150
SPCC70.05cR	CATAGCCTTTTCTAAGAGAT	
*tf2-12*F	AAGCATGTACCAGAGATAGG	150
*tf2-12*R	TGAATCACCTAGAAGAATTA	
*ung1*F	ACTTTGGAGAGTTCTTGGTT	150
*ung1*R	TGGAGTATGATGTGACCATG	

F = Forward; R = Reverse
